# Refugee Family Health Brokers’ (FHBs’) Experiences with Health Care Providers: A Thematic Analysis

**DOI:** 10.3390/ijerph20075381

**Published:** 2023-04-03

**Authors:** Bibhuti K. Sar

**Affiliations:** Kent School of Social Work and Family Science, University of Louisville, Louisville, KY 40208, USA; b.k.sar@louisville.edu

**Keywords:** family health broker (FHB), refugees, managing chronic health conditions, health care providers, health care barriers and challenges, cultural competence, humility, safety, thematic analysis

## Abstract

Background. The resettlement and post-resettlement quality of life of refugees is often marred by chronic health/mental health conditions. To adequately care for refugees suffering these conditions, a promising strategy is the use of refugee Family Health Brokers (FHBs). FHBs are safe and trusted family members functioning as intermediaries between one’s family and health care providers. Although FHBs are known to positively influence health care utilization in their families, little is known about them and this aspect of their family caregiving role and experiences, particularly with health care providers, necessitating further research. Methods. Fourteen Bhutanese and three Bosnian refugee FHBs participated in a 2-hr focus group discussing their experiences with health care providers after being surveyed about their FHB role. Results. Thematic analysis yielded five themes centered around perceptions, knowledge, communication, behavior, and responsibilities reflective of FHBs’ experiences, which can be understood as symptoms of existing structural inequalities. Conclusions. FHBs primarily conveyed problems, struggles, and dilemmas they experienced more so than rewarding aspects of being an FHB. Suggestions are provided on how to avert these negative experiences from occurring and becoming barriers to developing allyship with FHBs in the context of existing structural inequalities.

## 1. Introduction

Refugee resettlement and post-resettlement quality of life are directly affected by physical and mental health problems (e.g., diabetes, high blood pressure, heart disease, PTSD, depression, and anxiety disorders) [1,2,3,4]. Often identified during initial resettlement health screenings, these and other physical and mental health problems contribute to resettlement stress and impede successful resettlement.

Barriers to health care utilization are also a contributing factor. Frequently encountered barriers are lack of timely access to culturally and linguistically appropriate care and treatment, limited/lack of transportation, difficulty navigating through the health care bureaucracy, and high costs including inadequate/lack of health insurance coverage. Even when these barriers are removed, other challenges remain. Some common challenges faced are differing views held by refugees and health care providers about the causation of and treatment for physical and mental health problems [5], discomfort discussing physical and mental health histories (e.g., trauma) with health care professionals, concerns with being associated with the stigma attached to certain physical (e.g., HIV/AIDS) and mental health problems (e.g., depression or suicide), and fear of deportation if certain physical or mental health conditions were to be revealed [6,7].

A number of strategies have been employed to mitigate these barriers and challenges. One of the commonly utilized strategies is increasing basic health literacy, or “the degree to which organizations equitably enable individuals to find, understand, and use information and services to inform health-related decisions and actions for themselves and others” (p. 1) [8]. Health literacy has been found to be extremely low in vulnerable, medically underserved populations such as immigrants and refugees [9,10]. Health literacy programs are widely used and have been shown to increase knowledge of refugees’ medication use and understanding of health information through dissemination to a broader segment of the community (see for example, [11,12]).

A second strategy in use is that of community health workers (CHWs) or community health navigators (CHNs) who are employed within public health or primary care provider organizations serving marginalized populations [13]. CHWs/CHNs are thought to be better able to respond to the health needs of refugees and immigrants because they share similar cultural backgrounds, as they are typically lay community members with varying levels of academic or non-academic preparation who received training to provide health-related education and support for patients [14]. The value of using local CHWs/CHNs in the provision of basic health services in refugee camps overseas is widely acknowledged [15,16]. CHWs/CHNs in the United States have shown some promise in improving refugee and immigrant health and increasing their access to and integration into the American health care system [17].

Along with health literacy programs and use of CHWs/(CHNs), an alternative strategy proposed and introduced here that is relied on but not fully explored is Family Health Brokers (FHBs). An FHB is a safe and trusted family member who functions as an intermediary between one’s family and health care providers (i.e., doctors, nurses, counselors, pharmacists, physical/occupational therapists) to access the best care possible for their family member. Refugee FHBs function more than just as interpreters. They facilitate access to care (i.e., transportation); provide social and emotional support around the illness; track adherence to prescribed medical treatments; advocate, ask, and negotiate on behalf of their family member for services; are an ally to both the health care provider and their family member; and serve as a cultural bridge and mediator to meet their family member’s health care needs. FHBs are different from CHWs/CHNs in that they are more likely to have a more current and deeper knowledge of their family member’s health conditions, needs, and preferences, and thus can more directly communicate family members’ health concerns with health care professionals. Since health-seeking behavior typically occurs in the context of close family relationships outside of clinical and public health settings [18], employing FHBs may be as efficacious as employing a CHW/CHN in relation to improving refugees’ overall health/mental health outcomes.

### 1.1. Refugee History

The study reported here was devised to further understand the role of refugee FHBs. Refugee FHBs residing in a midsized southern city in the United States were invited and recruited with the assistance of refugee-serving organizations and refugee community leaders. Bhutanese (ethnic Nepali) and Bosnian refugee FHBs responded to the invitation and provided their input to further understand their experiences with health care providers. Before proceeding to a discussion of previous research in this area and the study methods used, a brief history of Bosnians and Bhutanese (ethnic Nepali) refugees is provided to contextualize the study findings.

Bosnian and Bhutanese (ethnic Nepali) refugees resettled in the United States, starting in the late 1990s and continuing throughout the 2000s. The resettlement of Bosnian refugees in the United States resulted from the Yugoslavian civil war (1992–1995). In 1992, the Yugoslavian republic, Bosnia and Herzegovina (Bosnia), with a population of 4.4 million which consisted of Bosniaks (Bosnian Muslims), Serbs, Croatians, and Yugoslavs, declared its independence with the aim of establishing an independent Bosnian nation consisting of a Bosniak (Bosnian Muslims) majority. In response, Bosnian Serbs retaliated by taking military action and engaging in “ethnic cleansing”—the systematic killings, torture, rape, and forcible displacement of Bosniak and Croatian civilians living in areas under Serb control. In July 1995, Bosnian Serb forces killed 8000 Bosniak men and boys in the town of Srebrenica. By the time of the signing of the Dayton Accords/peace agreement in 1995, one estimate placed the death toll at 100,000, with 80% of them being Bosniaks. Another estimate more than doubled the number killed, at 250,000, with 1 million people displaced and 1.3 million fleeing the war zone and the country [19,20].

Some 131,000 Bosnian refugees were initially admitted into the United States. A second wave of resettlement in the U.S. occurred in the late 1990s and early 2000s when Bosnians who had fled to neighboring European countries during the civil war were pressured to leave and return to their homeland or resettle in another country [20]. In resettlement, Bosnians were found to be struggling with loneliness and isolation due to loss of community, separation from family members, poverty, inadequate housing, culture shock, loneliness, psychic numbness, grief, nostalgia, and feelings of dejection, humiliation, inferiority, and feeling as if they belonged nowhere [21,22]. For Bosnian refugees’ unrecognized educational and professional credentials, their lack of proficient English language skills, and their perceived negative American attitudes towards them posed an immense challenge for their economic and social integration [20,23]. Bosnian refugees reported high levels of traumatic exposure to war and other traumatic stressors which were still associated with high severity of PTSD, symptoms of depression, and anxiety nearly a quarter of a century after the war [24].

They worried about adequate health care insurance coverage and choice and disliked the red tape of managed care and the long waits for appointments [25]. Health care interactions were characterized as more impersonal, less sensitive, and overly concerned with efficiency at the expense of physical and psychological comfort. Common were delays and long waits to receive care, and frustration about the process for accessing specialists in the United States, to name a few experiences and perceptions.

The migration of the Nepalese into Bhutan dates to the 17th and 19th centuries when Nepalese migrant workers came to Bhutan fulfilling a labor need as well as seeking employment opportunities. Over the centuries, they lived in peaceful co-existence with their Bhutanese neighbors, but for the most part did not integrate fully into Bhutanese culture, instead keeping to their culture, traditions, language, religion, and dress. Beginning in the 1980s, the King of Bhutan and the Bhutanese government began to be concerned over the growing Nepalese population in Bhutan, and the external influence of the Greater Nepal Communist Political Movement that was sweeping through Nepal and other countries where there was a Nepalese presence. In response, the King and the government created the “One Country/Nation, One People” initiative, also known as “Bhutanization”, which resulted in bans on Nepalese cultural, religious, and language practices and way of life such that teaching Nepalese in schools, practicing Hinduism, and wearing traditional Nepalese clothing in public were forbidden. Nepali-speaking schools, hospitals, and post offices were forced to close, and ethnic Nepali Bhutanese holding government positions were let go. In addition, citizenship verification tests were administered to root out non-Bhutanese citizens. In these actions, the King and government of Bhutan began to systematically dismantle and erase the culture of ethnic Nepali Bhutanese through enforcement of the majority Drukpa culture, dress, religion, and language on all Bhutanese regardless of their cultural heritage. Protests and anti-government attacks from ethnic Nepalese politicians in response to these actions resulted in forced displacement of more than 100,000 ethnic Nepalese into neighboring countries, mostly into Nepal. The government of Nepal was less than welcoming, as it was dealing with its own political and economic struggles, and confined the ethnic Nepalese from Bhutan to refugee camps in eastern Nepal, where they resided until Nepal and Bhutan finally agreed in 2006 on third-country resettlement, with ethnic Nepali Bhutanese being resettled in the United States, as well as in Canada, Australia, New Zealand, Denmark, and Norway. Some 86,000 Bhutanese refugees have resettled in the United States since late 2007 [26,27].

Bhutanese refugees identified their acculturation stressors as language barriers, loneliness, social isolation, depression, lack of interpreters when seeking health care and social services, and transportation problems in terms of access and reliability [28].

In the refugee camps, health care was directly associated with illness and the presence of symptoms. Remedies were initially sought through the family and traditional healers [28]. Bhutanese refugees were mistreated in the Nepalese health care system, often neglected from health care access and services because of their refugee status. Upon arrival to the United States after resettlement, study participants also reported experiencing challenges within the US health care system including cultural and linguistic barriers when interacting with medical interpreters during visits with their providers, as well as having inadequate time during the visit to fully express their concerns [29].

### 1.2. Previous Research

Few studies have explored experiences of refugee family health brokers. Although refugees perform many brokering functions, researchers have primarily examined two aspects: (1) interpretation—most commonly when family members are placed in the role interpreter; and (2) education—recruiting and training family members to serve as intermediaries. Banas et al. found that adolescent children of limited English-speaking parents served as interpreters in health care situations by performing such tasks as reading prescriptions and talking with doctors [18]. Katz noted that immigrant children served to both facilitate their families’ connection to health care providers and compensate for the lack of adequate language accommodations provided for their families by the health care system [30]. Hess et al. concluded that Burmese and Bhutanese adolescents were proud to be serving as intermediaries for their families, but it disrupted their daily lives because of having to navigate between both their home and American culture [31]. Leanza reported that health care providers found use of informal/family interpreters to be inadequate and problematic [32].

As per education, Roman et al. found that recruitment and enrollment of hard-to-reach African American, Latina, and Arab women for cancer screenings increased after they were recruited by family members trained and educated on the benefits of cancer screenings [33]. Chaoniyom et al. observed that Family Health Leaders (FHL) (defined as “representatives of families who take responsibility for the family members’ health” (p. 1039)) who were trained in knowledge, ability, leadership, and motivation to advance health promotion activities were the “key persons to bring good health to family members” (p. 1047) [34].

### 1.3. Purpose of the Study

The findings of the previous research suggest that refugee family members, particularly children and adolescents, should not function as intermediaries. Young people are conflicted and negatively impacted when placed in such roles. On the other hand, adults serving as intermediaries, other than as interpreters, have benefits such as increasing participation in health screenings, and possibly mitigating one or more barriers to health care utilization. Thus, this study is undertaken to further explore the experiences of refugee family health brokers. A better understanding can potentially be helpful in addressing some of the barriers and challenges previously mentioned. The findings may ultimately inform better management of health conditions while saving money, time, and effort.

## 2. Materials and Methods

This research was part of a broader effort/study (approved by IRB #18.0645) to better understand refugee family health broker roles in the provision of health and mental health care. The setting for the study was a midsized southern city in the United States (as stated previously) with a long history of refugee resettlement. Participants who (1) were 18 years of age or older, (2) spoke English, and (3) were serving as an intermediary/FHB were recruited through refugee-serving organizations with the assistance of refugee community leaders. Participants were consented prior to completing a brief survey and taking part in a 2-h focus group, framed as a listening session. They completed the survey by providing demographic information and answering questions about their family member’s health, caregiving activities they performed, and their confidence in communicating with health care providers. Their focus group responses were guided and prompted by “*We are interested in hearing about your experiences with healthcare providers (i.e., doctors, nurses, counselors, medical assistants, etc.) in getting care for your family member*”. A total of three focus groups—two with Bhutanese (*n* = 8) (*n* = 6) and one with Bosnians (*n* = 3)—were conducted by the author, assisted by a research coordinator who took notes and audiotaped the sessions. Participants were given a USD 10 gift card as compensation for their time at the end of the focus group.

### Analysis

The survey data were entered into SPSS v26 and analyzed using descriptive statistics. The audiotaped focus group responses were transcribed into one document, and the notes taken during the focus group and a summary of them were transcribed into a separate document by the research coordinator. The six steps of thematic analysis—familiarization, generating codes, generating themes, reviewing themes, defining/naming themes, and creating the report [35]—were applied to the transcribed focus group responses and research coordinator notes and summaries to identify themes indicative of FHBs’ experiences.

Given the exploratory nature of this study, thematic analysis was selected to provide a rich description of FHBs’ experiences. An essentialist/realist frame was adopted in carrying out the data analysis by focusing on reporting experiences, meanings, and reality of the focus group participants. This consisted of taking an inductive approach to identify themes strongly linked to the data, data-driven coding, and identifying themes at the semantic level that involved description and interpretation theorized in relation to previous literature [35].

First, as part of the familiarization process, the focus group transcripts were read and reread several times while jotting down initial ideas about the data. Then, initial codes were generated and assigned by systematically going through the transcripts line by line from beginning to end. At this point, the research coordinator notes and summaries were introduced to the analysis, checked, and reviewed against the codes developed by the author. As a result, the initial codes generated by the author were revised to reflect the inclusion of the researcher notes and summaries into the analysis. Next, with this revised set of codes, similar codes were grouped into categories and collated into potential themes. These themes were checked and reviewed for how well they reflected the codes and fit the original data from which the codes were developed. The themes were named and defined. Extracts from the data that reflected the developed themes were identified and were related back to the primary purpose of the research, which was to describe the experiences of FHBs.

## 3. Results

### 3.1. Demographics

As indicated in Table 1, the focus group participants were from the ethnic Nepalese Bhutanese and the Bosnian refugee communities. The 17 FHBs consisted of both men (53%) and women (47%) ranging in ages from 29 to 63 (average age 42). They identified as either Asian (82%) or White (18%). Slightly more than half of the FHBs (52.9%) were employed fulltime, while an equal percentage were employed parttime (23.5%) or unemployed (23.5%). Their religious background was Hindu (76.5%), Muslim (17.6%), or Kirati (5.9%). They came to the United States as refugees from Bhutan (82.4%) or Bosnia (17.6%).

A majority (70.6%) of the FHBs lived with a relative with a chronic health condition, 17.6% did not live with a relative, and 11.8% did not report their place of residence. Sixty-three percent of the FHBs had lived with their relatives for more than 15 years. Twenty-five percent have had with their relatives 7–12 years, and 13% had lived with their relative 4–6 years. Slightly more than one third of the subjects (35%) were FHBs to their father. A comparable percentage (36%) were FHBs to either their husband, mother, sister, daughter, son, father-in-law, or a nonrelative. These family members with a chronic health condition were aged 12 to 73 (average age 63), and a majority were male (86%).

### 3.2. Family Member’s Health

FHBs reported that their family members suffered from a range of illnesses including cancer, diabetes, high blood pressure, high cholesterol, and chronic back pain. There had been very few emergencies room visits (11.8% reported one visit, and 5.9% reported two visits) and hospitalizations (5.9% reported one, and 5.9% reported two) in the past three months. Nearly half (47.1%) of the FHBs rated their family member’s overall health as poor, 41.2% as fair or good, and 11.8% as “don’t know”.

### 3.3. Actions Performed as FHBs

FHBs were asked to indicate how often they performed a set of caregiving activities and tasks from a list which was generated from a review of the literature and discussions held with refugees, refugee community leaders, and health care providers. As can be seen in the last column in Table 2, these activities and tasks correspond to one of six domains of family caregiving roles identified by researchers and practitioners [36]: assistance with household tasks (AHTs) (e.g., helping with bills or transportation); self-care, supervision, and mobility (SCSM) (e.g., bathing or help getting around); emotional and social support (ESS) (e.g., helping the family member manage emotional responses); health and medical care (HMC) (e.g., encouraging treatment adherence); advocacy and care coordination (ACC) (e.g., facilitating understanding of provider, family, and person, or making appointments); and surrogacy (SGC) (e.g., participating in treatment decisions). Eight of the 20 tasks on this list were specific brokering tasks (those falling into the ACC domain). Between 35.3% and 58.8% refugee FHBs indicated that they performed ACC activities “all the time”.

### 3.4. Confidence in Communicating with Health Care Providers

Only 35.3% of the FHBs stated that they were totally confident engaging service providers to ask about concerns they had about their family member’s illness, 47.1% said they could openly discuss any personal problems that may be related to the family member’s illness, and 41.2% stated that they can work out differences with their relative’s service providers.

### 3.5. Focus Group Results

Thematic analysis of the focus group responses resulted in the following themes: (1) perceptions: mixed feelings, ulterior motives, and distant interactions; (2) knowledge: lack of cultural knowledge is a path to cultural malpractice; (3) communication: miscommunication can result in harmful health care; (4) behavior: sidestepping rules and protocols is at times necessary; and (5) responsibilities: there is no winning trying to take care of everyone’s needs.

#### 3.5.1. Theme 1: Perceptions: Mixed Feelings

This theme is a description of the mixed feelings and emotions experienced as a result of FHBs’ interactions with health care providers. On the one hand, they perceived that they and their family members were cared for, as reflected in the following statements.

[With a past health care provider] *“I had a really good lady. She gave me more directions. She was calling every month. I never met her, but she did a lot of good things for us. She found a really good clinic for me. She set up the insurance.”*

*“They are really nice. One nurse clocked out and spent one hour with my child so that I could take a break. I call her ‘my mom.’ She has a special place in my heart.”*

*“I like health care services in”* [city that they live in].

On the other hand, they indicated:

*“I don’t know what they* [doctors] *think of us.”*

*“They just go from office to office. They don’t even touch the patient. They just say ‘You look fine. Keep it up.’ They give you medication and tell you to follow-up in two months.”*

*“They don’t have time for patients.”*

*“They just ignore us.”*

*“Yes, sometimes they will* [take advantage of us]. *It happens more to the older generation* [of …] *than the younger generation.”*

*“A lot of them just come to work to make money.”*

*“The more patients they have, the more money they are going to get.”*

#### 3.5.2. Theme 2: Knowledge: Lack of Cultural Knowledge Is a Path to Cultural Malpractice

This theme captures family health brokers’ concerns about and consequences of health care providers’ lack of cultural knowledge and practices in the delivery of health care. Some FHBs observed health care providers are either unaware or not knowledgeable about how to communicate a diagnosis of serious illness and recommended treatment in a culturally sensitive manner. Others expressed that their family member’s reported symptoms were dismissed, ignored, or were indicated as imagined. Some felt excluded from their family member’s medical appointments, even though their presence eased their family member’s reluctance and discomfort talking about their health problems with people outside of the immediate family, including health care providers. All of this was expressed as tantamount to potential malpractice, as expressed below:

*“American doctors are more direct with telling you that you have cancer.”*

*“In* [my country] *doctors are not so direct with you. My mother almost died from just hearing the news from the doctor that she had cancer.”*

*“They should share the information with the people because they have a right to know what is going on with them. With things like cancer, they need to talk to family members first to decide whether or not to discuss it with the patient or not.”*

*“I need the doctor to tell me what is wrong with her rather than just relying on a Google search about her condition. You have to be rude sometimes. I feel that I have to say something.“*

*“I will say that some of the nurses need to be trained better. They say they do more than they are actually doing. I told them things that they didn’t even know about.”*

#### 3.5.3. Theme 3: Communication: Miscommunication Can Result in Harmful Health Care

Many first-generation refugees with health problems face language and literacy barriers and thus are not able to communicate and act on their health concerns.

As one participant explained, “They can call 911, but it is difficult for them to explain what is going on. They wait until a family member can call. That can be a real problem.” Thus, interpreters and proper and accurate interpretation are key to family members receiving the best health care, but the system of interpreters and interpretation posed challenges for participants and their family members. Miscommunication was a common experience reported, sources of which included problems with the type of interpretation method used (in-person versus phone interpretation), language and dialect differences, lack of access to the same interpreter, and varying level of interpreter expertise. Further, some participants expressed concerns over privacy and frustration of not being able to interpret for their family member, while others felt the doctor trusted the interpreter more than them to convey medical information to their family member. As expressed below, these experiences were alarming because they have a direct impact on the health care their family members received.

*“We don’t have any privacy concerns between family members. We can’t tell everything 100% to the phone interpreter.”*

*“When we go to the doctor, we need the interpreter. The interpreter can tell us what the doctor said so it can be explained to anybody. An in-person interpreter is better than a phone interpreter.”*

*“The in-person interpreter is the best [option]. It is really difficult to understand some interpreters over the phone. They don’t really converse in a way we understand.”*

*“Due to phone interpreters speaking with a different dialect, it is difficult to understand each other. Miscommunication happens due to using different words for the same thing.”*

*“Yes, they sometimes miss the medication names. That is a problem.”*

*“Sometimes the interpreter misinterprets what the doctor says. Big words are often wrongly interpreted. This can have harmful effects. The interpreter needs to tell the doctor ‘I need to clarify the word.’ The interpreter has to take care of that part.”*

*“They* [doctors] *want to hear every* [health problem] *episode, but the interpreters are not prepared to tell everything.”*

*“It is better for us to be the interpreter, but many times the health provider will not allow us to do the interpretation for our family member.”*

#### 3.5.4. Theme 4: Behavior: Sidestepping Rules and Protocols Is at Times Necessary

At times participants found themselves having to sidestep or bend rules in their role as an intermediary in order to ensure access and proper care for their family member. Other times they found alternative ways to share their input with the health care providers, as expressed below.

*“Yes, but sometimes we go into the room anyway.”* [in response to when they are told that the family member cannot be in the examining room]

*“The home health care rules prohibit me from getting paid for caring for my own relative, so I swap out caring for my friend’s relative and they do the same for my relative.”*

*“Sometimes writing the problem in English on a piece of paper and handing it to the doctor is a way of doing it.”*

#### 3.5.5. Theme 5: Responsibility: There Is No Winning in Trying to Take Care of Everyone’s Needs

Participants expressed that serving as an intermediary is full of competing responsibilities with little time for self-care. As one participant indicated, “*The joke is that I take better care of my vehicle than I do of myself*”. Participants felt torn and conflicted as to how to be available and be there for everyone.

*“She* [the other child] *is a bit jealous of the attention we pay to our… child* [who is ill]. *It is really hard to explain the extra attention paid to …child”* [who is ill]

*“He* [husband] *needed too much attention from me.”*

*“There is a fear, always. We lose on both sides. If we do not take the family member to the doctor, we might lose them [death]. If we do not work, we might lose our job. It is hard to balance. Both sides there is losing.”*

## 4. Discussion

In this study five themes reflective of refugee FHBs’ experiences were identified through thematic analysis. They should be viewed in the context that the study consisted of a small sample of self-selected refugees from two cultural groups who self-reported their FHB experiences. FHBs asked to discuss their interactions with all types of health care providers rather than a specific category of health care providers (i.e., doctors, nurses, pharmacists, etc.), so these findings should not be blanketly applied to understanding experiences of *all* refugees with *all* or a specific group of health care providers. Additionally, the results should be applied and interpreted within the context of limitations of thematic analysis regarding its flexibility as a method, and its limited interpretive power [35].

Overall, the findings corroborate the experiences of other refugees receiving health care while expanding understanding of FHBs’ experiences. Refugee FHBs’ perceived treatment in the provision of health care formed their judgement of health care providers (theme 1). Previous studies have reported similar findings. Somali refugee women expressed fear and mistrust toward Western health care, health care providers, and diagnostic tests but also acknowledged positive encounters with health care providers who were polite, attentive, and expressed interest in their lives and were perceived to be engaging in skilled caring practices [37]. Positive perceptions of health care providers were an influential factor in traumatized Yazidi refugees’ utilization and perception of health care [38]. This underscores not only how important it is for refugees to have a positive experience with health care [39] but also for FHBs whose perceptions may influence the extent of health care utilization by their family members with chronic health conditions. FHBs’ perceptions of health care providers may influence their family member’s use of health services.

Previous research findings echo FHBs’ observations and concerns about health care providers’ gap in cultural knowledge and practices (theme 2). It has been reported that some health care providers overestimated their cultural knowledge and expressed confidence in their ability to meet the needs of multicultural clients even though they had not participated in formal cross-cultural training [40]. It has been suggested that if health care providers were “diversity sensitive” in their interactions with their refugee patients, many of the barriers to health care could be resolved [41].

FHBs’ dissatisfaction with and warning about harm resulting from miscommunication (theme 3) are corroborated by interviews with refugees which revealed that they were concerned about interpreters not maintaining confidentiality, not understanding medical terminology, and not speaking the same dialect [42]. Miscommunication was found to be a barrier to managing medications [43] and that it can harm refugees’ health [33]. Miscommunication concerning medical procedures resulted in perceived unsatisfactory care [44].

FHBs sidestepping rules and protocols (theme 4) has not been reported in previous studies with refugees. In the context of challenges of understanding and navigating the U.S. health care system, sidestepping rules and protocols is not surprising. It is understandable that any family member, refugee or not, would also take steps as FHBs did to communicate important health information to health care providers, participate in and monitor care being provided, and ensure their family member received proper care.

FHBs “may be motivated by the desire to do one’s job better or to do what one believes to be appropriate in a given situation” (p. 7) [45]. This may be understood as instances of prosocial rule breaking (PSRB). PSRB is defined as “any instance where an employee intentionally violates a formal organizational policy, regulation, or prohibition with the primary intention of promoting the welfare of the organization or one of its stakeholders” (p. 6) [45]. PSRB has been advocated in health care systems to challenge and eliminate existing rules and regulations that pose a barrier to better health care practice [45].

The sense of a “no-win” situation expressed by FHBs (theme 5) is consistent with research on family caregiver experiences. Caring for a family member with a chronic health condition can have several emotional, social, and physical impacts on the caregiver. Caregivers can struggle with balancing and meeting their responsibilities to others or paying attention to their needs. The burden of caregiving comes with feelings and emotions such as sadness and depression, worry, fear, stress, anxiety, and loss as well as feelings of resentment toward the ill family member. The emotional tensions and distress can be an outcome of the “spillover effect” [46] of caring for ill family members. The effects can be physical, psychological, as well as affecting work/jobs, finances, social activities, family relationships, and self-care. All these effects can vary by the existing type of relationship with the ill family member, the family member’s health condition, the illness the family member is experiencing, needs associated with managing the illness, family, and external supports and resources available, and cultural and intergenerational roles and expectations [47].

The five themes discussed here may seem on the surface as unremarkable findings which are just ordinary, typical, and common responses that would be expected from not only refugees but non-refugees as well. After all, who has not had an interaction with a health care provider where they felt misunderstood and questioned the health care provider’s interest and investment in them and their health concerns? However, examined from a slightly different perspective, these findings are symptoms of the much deeper structural inequalities that FHBs and their family members must navigate through in accessing health care on a regular basis. Structural inequities (e.g., encompassing policies, laws, governance as well as culture, race, ethnicity, gender or gender identity, class, or sexual orientation) refer to “the systematic disadvantage of one social group compared to other groups with whom they coexist that are deeply embedded in the fabric of society” (p. 100) [48]. They are the purveyors of “systematic disadvantages, which lead to inequitable experiences of the social determinants of health and ultimately shape health outcomes” (p. 100) [48]. In this study, the experiences of FHBs—language barriers, inadequate interpreter services, and othering—are all part of the nexus of structural inequalities. Policy makers need to implement policies that dismantle the underlying structural arrangement that keeps these problems in place. Such actions will not only benefit FHBs and their family members but also unhinge the structural arrangements under which health care providers find themselves operating knowingly or unknowingly. A step in that direction, for instance, would be policies and programs increasing the racial, ethnic and culturally diverse make-up of the health care workforce who provide refugee health care. This would require creating pathways and providing resources (e.g., scholarships) for these members to have the means to pursue a career in the health professions to meet this need.

## 5. Conclusions

In addition to addressing structural inequities, these findings also suggest pursuing more immediate changes including several ways that refugees can be supported and validated as FHBs. First, they need to be acknowledged and feel valued for the important role they play in ensuring that their family members receive the care they need. One way to do this is to formalize FHBs’ role and compensate them for their time, such as the system in place for employing CHWs/CHNs. Second, FHBs should be asked for input about how to engage their family members during the provision of services. This can begin by health care professionals acknowledging FHBs as allies and partners in the care of their patients. Third, to fully engage FHBs as partners, health care providers should be trained in the provision of equal and humane treatment which is more important than just acquiring culture-specific information [49]. There is a need for health care providers to engage in professional development that includes bringing awareness to one’s prejudices as well as focusing in on recognizing racism, power imbalances, and majority culture biases [42] that factor in health care interactions. This needs to be included as part of the initial training and education curricula of health care providers. Some health care providers’ (i.e., medical students) curricula have been shown to be lacking in content related to refugees [50]. It has been recommended, for example, that medical students receive training with a focus on improving self-perception of cross-cultural knowledge, communication, in addition to physical exam skills [51].

Furthermore, health care providers should be expected to go beyond achieving cultural competence to demonstrating the integration of cultural humility and cultural safety principles in their practice. Cultural humility is incorporating “a lifelong commitment to self-evaluation and self-critique, to redressing the power imbalances in the provider-client dynamic, and to developing mutually beneficial and non-paternalistic clinical and advocacy partnerships with communities on behalf of individuals and defined populations” (p. 117) [52]. Also critical is cultural safety, which is defined as “a focus for the delivery of quality care through changes in thinking about power relationships and patients’ rights” (p. 493) [53]. This has been expanded to also include the role of health care organizations “to acknowledge and address their own biases, attitudes, assumptions, stereotypes, prejudices, structures and characteristics that may affect the quality of care provided” (p. 14) [54].

FHBs should be provided with information on understanding and navigating the health care system in both written and oral forms [51]. Increasing their health literacy will be empowering and may lessen instances of discrimination and marginalization that are directed at them [51]. FHBs need support to manage stress associated with the “push” and “pull” of attending to family members’ needs while not forgetting to take care of themselves. The dynamics that sometimes occurs in the provider–FHBs–interpreter communication is another source of stress that requires further study.

In conclusion, FHBs primarily described problems, struggles, and dilemmas they experienced more so than rewarding aspects of being an FHB. A set of themes centered around perceptions, knowledge, behavior, communication, and responsibilities reflective of FHBs’ experiences were identified. More studies are needed to better understand how these experiences may factor into promoting or impeding FHB–health care provider interactions and overall health care utilization.

## Figures and Tables

**Table 1:** Demographic characteristics of focus group participants.

Focus Group Participant	Gender	Age	Employment	Country of Origin	Race	Religion	Relative with Chronic Health Condition Live with You?	How Long has the Relative Lived with You?	Who Is the Relative?
1	Male	40	Fulltime	Bhutan	Asian	Hindu	No	N/A	Father
2	Male	29	Parttime	Bhutan	Asian	Hindu	Yes	7–9 years	Father
3	Male	30	Fulltime	Bhutan	Asian	Hindu	Yes	Not stated	Father
4	Male	39	Parttime	Bhutan	Asian	Kirati	Yes	4–6 years	Father
5	Female	39	Fulltime	Bhutan	Asian	Hindu	No	N/A	Other relative
6	Male	63	Unemployed	Bhutan	Asian	Hindu	Yes	15+ years	Wife
7	Male	34	Parttime	Bhutan	Asian	Hindu	Yes	15+ years	Mother
8	Male	43	Fulltime	Bhutan	Asian	Hindu	No	N/A	Father
9	Female	49	Fulltime	Bhutan	Asian	Hindu	Yes	15+ years	Son
10	Male	30	Fulltime	Bhutan	Asian	Hindu	Yes	Not stated	Not stated
11	Male	54	Fulltime	Bhutan	Asian	Hindu	Yes	Not stated	Mother
12	Female	29	Fulltime	Bhutan	Asian	Hindu	Not stated	Not stated	Not stated
13	Female	55	Unemployed	Bhutan	Asian	Hindu	Yes	15+ years	Father
14	Female	34	Fulltime	Bhutan	Asian	Hindu	Yes	10–12 years	Father-in-law
15	Female	59	Unemployed	Bosnia	White	Muslim	Yes	Not stated	Husband
16	Female	54	Unemployed	Bosnia	White	Muslim	Yes	15+ years	Husband
17	Female	34	Parttime	Bosnian	White	Muslim	Yes	Not stated	Daughter

**Table 2:** Percentage of FHBs indicating actions performed “all the time” on behalf of their family members.

Action		% FHBs Indicating “All the Time”	Family Caregiver Domain *
1	Keeping track of the medical doctor’s instructions/orders for the family member	64.7%	HMC
2	Driving the family member to his or her appointment with service providers	64.7%	AHT
3	Advocating for relative’s health care needs with service provider	58.8%	ACC
4	Keeping track of instructions given to family member by service providers (i.e., nurse, psychiatrist, or counselor)	58.8%	HMC
5	Going with family member to their doctor’s appointments	58.8%	AHT
6	Picking up prescriptions/medicines from the pharmacy for family member	58.8%	HMC
7	Expressing concerns about my relative’s health to health care provider	52.9%	ACC
8	Mediate/intervene to make sure that my relative’s health care needs are understood	52.9%	ACC
9	Letting the doctor know of family member’s health concerns	52.9%	ACC
10	Reminding family member to take their medication	52.9%	HMC
11	Providing emotional support to family member when accompanying them to appointments	47.1%	ESS
12	Speaking up for their family member with providers when relative does not express all their health concerns	47.1%	ACC
13	Making health care decisions for family member when they cannot decide what to do about a health condition or illness	47.1%	SGC
14	Clarifying information between service provider and family member	47.1%	ACC
15	Making effort to ensure that their family member understands their illness	41.2%	HMC
16	Scheduling doctor’s appointments for their family member	41.2%	ACC
17	Taking part in making decisions about family member’s overall health care	41.2%	SGC
18	Making effort to ensure that family member understands the medications they are taking	41.2%	HMC
19	Serving as interpreter/translator with service providers	35.3%	ACC
20	Making effort to ensure that family member understands treatments that he or she is having, undergoing, or needs	35.3%	HMC

* AHT: assistance with household tasks; SSM: self-care, supervision, and mobility; ESS: emotional and social support; HMC: health and medical care; ACC: advocacy and care coordination; SGC: surrogacy.

## Data Availability

The data are not publicly available due to privacy, ethical, and IRB requirements.
